# Complete mitochondrial genome sequences for Crown-of-thorns starfish *Acanthaster planci *and *Acanthaster brevispinus*

**DOI:** 10.1186/1471-2164-7-17

**Published:** 2006-01-27

**Authors:** Nina Yasuda, Masami Hamaguchi, Miho Sasaki, Satoshi Nagai, Masaki Saba, Kazuo Nadaoka

**Affiliations:** 1 Mechanical and Environmental Informatics, Graduate school of Information Science and Engineering, Tokyo Institute of Technology, 2-12-1 Ookayama, Meguro-ku, Tokyo 152-8552. Japan; 2 National Research Institute of Fisheries and Environment of Inland Sea, Fisheries Research Agency, 2-17-5 Maruishi, Hatsukaichi, Hiroshima 739-0452, Japan; 3 581-60 Sakura-cho, Matsuzaka, Mie 515-0071, Japan

## Abstract

**Background:**

The crown-of-thorns starfish, *Acanthaster planci *(L.), has been blamed for coral mortality in a large number of coral reef systems situated in the Indo-Pacific region. Because of its high fecundity and the long duration of the pelagic larval stage, the mechanism of outbreaks may be related to its meta-population dynamics, which should be examined by larval sampling and population genetic analysis. However, *A. planci *larvae have undistinguished morphological features compared with other asteroid larvae, hence it has been difficult to discriminate *A. planci *larvae in plankton samples without species-specific markers. Also, no tools are available to reveal the dispersal pathway of *A. planci *larvae. Therefore the development of highly polymorphic genetic markers has the potential to overcome these difficulties. To obtain genomic information for these purposes, the complete nucleotide sequences of the mitochondrial genome of *A. planci *and its putative sibling species, *A. brevispinus *were determined and their characteristics discussed.

**Results:**

The complete mtDNA of *A. planci *and *A. brevispinus *are 16,234 bp and 16,254 bp in size, respectively. These values fall within the length variation range reported for other metazoan mitochondrial genomes. They contain 13 proteins, 2 rRNA, and 22 tRNA genes and the putative control region in the same order as the asteroid, *Asterina pectinifera*. The A + T contents of *A. planci *and *A. brevispinus *on their L strands that encode the majority of protein-coding genes are 56.3% and 56.4% respectively and are lower than that of *A. pectinifera *(61.2%). The percent similarity of nucleotide sequences between *A. planci *and *A. brevispinus *is found to be highest in the *CO2 *and *CO3 *regions (both 90.6%) and lowest in *ND2 *gene (84.2%) among the 13 protein-coding genes. In the deduced putative amino acid sequences, *CO1 *is highly conserved (99.2%), and *ATP8 *apparently evolves faster any of the other protein-coding gene (85.2%).

**Conclusion:**

The gene arrangement, base composition, codon usage and tRNA structure of *A. planci *are similar to those of *A. brevispinus*. However, there are significant variations between *A. planci *and *A. brevispinus*. Complete mtDNA sequences are useful for the study of phylogeny, larval detection and population genetics.

## Background

The crown-of-thorns starfish, *Acanthaster planci *(L.), is a typical reef coral predator, which sometimes causes population outbreaks and destroys coral reef communities. The activities of this starfish have been responsible for causing extensive coral mortality in a large number of coral reef systems throughout the Indo-Pacific region [1]. Major outbreaks involve large numbers of starfish and widespread coral destruction and consequently they can change the fauna and topographic nature of coral reef communities [1,2].

In Australia, Kenchington [3] hypothesized that outbreaks (termed primary) started and in turn initiated a cascade of additional outbreaks (termed secondary). Similarly, the Great Barrier Reef (GBR) in Australia and the Ryukyu Islands in Japan have experienced two distinct series of outbreaks of the crown-of-thorns starfish [2]. Once the unpredictable primary outbreak occurs, this large population of adult *A. planci *that reproduces enormous amounts of eggs can lead to secondary outbreaks.

The high fecundity and long pelagic larval stage [4] of *A. planci *imply that the dispersal and recruitment of larvae play an important role in the occurrence of primary and secondary outbreaks [5]. The lack of knowledge of its larval dispersal has limited the understanding of its population dynamics. Some hydrodynamic models have been developed to describe larval dispersal around the GBR [6,7]. These authors suggested that larval dispersal was not random and provided probabilistic routes for dispersal through the GBR. They also claimed that some larvae might be retained on the natal reef. Verifying the larval dispersal route predicted by these hydrodynamic models is essential. However, there are two difficulties in the study of larval dispersal and recruitment of *A. planci *from a biological perspective: 1) the morphological appearance of *A. planci *larvae is so similar to those of other asteroid species [4], hence, it is extremely difficult to differentiated *A. planci *larvae from other asteroid larvae. Recent concern about pelagic larvae dispersal emphasizes the need for accurate larval identification. 2) There have been no useful tools to reveal the reef-connectivity among reefs and autochthonous recruitment of *A. planci *larvae. Therefore, the development of highly polymorphic genetic markers such as mitochondrial sequences or nucleic microsatellites has been eagerly desired. To resolve the former problem, Evans [8] developed a Polymerase Chain Reaction (PCR)-based identification tool for other asteroid species larvae (excluding *A. planci*) and showed the PCR-RFLP assay is a useful tool for larval identification of asteroid larvae. The complete or partial mtDNA sequences of many metazoans are now available [9]. Having a relatively rapid evolutionary rate and lacking intermolecular genetic recombination, these data on mtDNA sequences have been utilized to identify invertebrate larvae [10] or conduct the population genetic study at various taxonomic levels [11]. The genetic information can be applied to overcome both these difficulties in the study of larval dispersal of *A. planci*.

In this study, we determined the complete sequences of the mitochondrial genome of *A. planci *and its sibling species, *Acanthaster brevispinus *and compared in detail the full sequences of both species and a third asteroid species, *Asterina pectinifera*. Also, we describe some characteristics of both mitochondrial genomes of *A. planci *and *A. brevispinus*.

## Results and discussion

### General Features

The complete mtDNA sequences of *A. planci *and *A. brevispinus *are 16,234 bp and 16,254 bp in size, respectively; both fit within the length variation range reported for other metazoan mitochondrial genomes [9]. They were submitted to the DDBJ with Accession No. AB231475 (*A. planci*) and AB231476 (*A. brevispinus*). They contain 13 proteins, 2 rRNA, and 22 tRNA genes [see additional file 1] in the same order as another asteroid species, *Asterina pectinifera *[12] [NCBI: NC-001627]. Both in *A. planci *and *A. brevispinus*, the L strand encodes most of the proteins including 10 proteins, 11 tRNA and 12S rRNA genes, whereas the H strand includes the remaining three proteins (*ND1*, *ND2*, and *ND6*), 11 tRNA and *16S rRNA *genes [12].

### Base composition and codon usage

Animal mitochondrial genomes sometimes deviate from a random nucleotide usage, which may be due to the directional mutation pressure and the non-asymmetrical base composition of both the strands [13]. The A + T contents of *A. planci *and *A. brevispinus *on the L strand are 56.3% and 56.4%, respectively, and both are lower than that of *A. pectinifera *(61.2%). This result comes from the fact that the third codon position of the protein-coding gene in *A. pectinifera *prefers T more than those of *A. planci *and *A. brevispinus*, particularly in the synonymous codons of Phe (UUU), Leu (CUU), Ile (AUU), Ser (UCU) and Asn (AAU) [see additional file 2]. The mtDNA sequences of *A. planci *and *A. brevispinus *have A-bias on the first and second codon positions of protein-coding gene and rRNA genes. There is no significant difference in the base composition of tRNA genes between the *Acanthaster *species and *A. pectinifera *[see additional file 3].

Asakawa *et al. *[13] demonstrated the bias of a strand-specific nucleotide composition in the mtDNA of *A. pectinifera *that the L strand prefers to use A or C while the H strand prefers to use G or T in the third codons. This asymmetric distribution of G and C between the two complementary DNA strands can be easily measured with the following formulae [14]; GC-skew = (G-C)/(G+C); AT-skew = (A-T)/(A+T) where C, G, A and T are the occurrences of the four bases. The skew is likely to be most pronounced at the third codon positions of each strand, where any mutational change is synonymous and not subject to selection pressure [15]. Thus we calculated the GC-skew and AT-Skew using the third codon positions of each strand. The L strand prefers to use A or C and H strand prefers to use G or T in both *Acanthaster *species, clearly showing strand-specific bias of the nucleotide compositions. The three asteroid species show almost the same tendency of skew, the skew in other five echinoderms (*Cucumaria miniata *(holothuroid), *Arbacia lixula *(echinoid), *Ophiopholis aculeata *(ophiuroid) and *Ophiura lutkeni *(ophiuroid)) showed similar bias of skew, although the degree of skew is slightly different among species examined except for *Florometra serratissima *(crinoid) (data not shown). In *F. serratissima *mtDNA, the L strand prefers to use G or T and the H strand prefers to use A or C. The pattern of the bias of the skew is likely to be conserved in asteroid species, although the strand-specific bias is not a general rule in echinoderms [16].

The strand specific bias of the A + C content of protein-coding genes is also seen. In short, the A + C content of the H strands in *A. planci *and *A. brevispinus *are 31.4% and 30.9 %, respectively, whereas those in the L strands are 57.3% and 57.6 %, respectively. These values are similar to those of *A. pectinifera *(34.1% for the L strand and 56.5 % for the H strand).

### Nucleotide and amino acid differences in protein genes

As previously mentioned, the identified protein-coding genes of the *A. planci *and *A. brevispinus *mtDNA are similar with those of *A. pectinifera *[12] and of other metazoan mtDNA genomes [9]. Most of the protein-coding genes in these species are identified by sequence comparison with *A. pectinifera *except for *ND5 *and *ND3 *genes. Alignment is difficult to perform because size variations occur near their 5' ends. The 5' ends of *ND3 *and *ND5 *genes in these two species are inferred from the eligible termination codons without overlapping the preceding gene. In the sequence comparison between *A. pectinifera *and *A. planci*/*brevispinus*, significant changes in the number of codons are observed in *ND3 *and *ND5 *genes between the *Acanthaster *species and *Asterina*. The nucleotide sequence of *ND3 *gene in *A. pectinifera *is 18 bp shorter than those of both *Acanthaster *counterparts. On the other hand, both *Acanthaster ND5 *gene nucleotide sequences are 10 bp shorter than that of *A. pectinifera*. In both of these cases, most of the deletions are observed near 3' end of each gene.

For the given genes, nucleotide and amino acid differences are compared in the protein-coding genes [see additional file 4]. The percent similarity of nucleotide sequences between *A. planci *and *A. brevispinus *is found to be highest in the *CO2 *and *CO3 *regions (both 90.6%) and lowest in *ND2 *gene (84.2%) among the 13 protein-coding genes. In the amino acid sequences, *CO1 *gene is highly conserved (99.2%), and it seems that *ATP8 *has evolved faster than any of the other protein-coding genes (85.2%). Amino acid sequences are generally more conserved than nucleotide sequences because of the functional constraints. Therefore, most of the identity values of amino acid sequences are higher than those of nucleotide sequences or almost the same except for *ATP8 *gene. The percentage similarity of amino acid sequences in *ATP8 *gene is significantly lower than those of nucleotide sequences between *Acanthaster *species and *A. pectinifera *and also slightly lower even between *Acanthaster *species. The pairwise comparison of the *ATP8 *gene among *A. planci*, *A. brevispinus *and *A. pectinifera *has revealed that the nucleotide substitution rates of nonsynonymous sites (first + second codon positions) are almost the same as those of synonymous sites (third position) (data not shown), suggesting the cause of this improbable phenomenon. A similar result in ATP genes was reported in sea urchins [17] and they showed that the nucleotide substitution rates of nonsynonymous sites are not significantly lower than that of synonymous sites. These data clearly indicate a relaxation of the functional constraints at the amino acid level. They found that the same sequence motif (V/M)PQL(X4)(W/F) is evolutionarily conserved from yeast to mammals at the N-termini of the proteins. When the deduced amino acid sequences were analysed with a computer aided method, the predicted hydrophatic profiles for the corresponding proteins are completely super-imposable. De Giorgi *et al*. [17] stated that in spite of the divergence observed in the primary structure of the genes, the role played may be the same. On the other hand, the loss of *ATP8 *has occurred several times in the evolutional process of metazoans [18]. As De Giorgi *et al. *[17] suggested, the C-terminal of *ATP8 *might evolve extremely rapidly and in a phylum-specific manner.

The *ATP8 *and *ATP6 *genes of starfish overlap each other by 16 nucleotides with a -1 frame shift. A similar overlap is found among metazoan mitochondria, although the extent of overlap varies [19]. Thus, it is easily expected that the functional constraints of *ATP8 *gene are not so large and the gene could evolve faster than other protein-coding genes. However, further work is required to clarify the causal relationship in the lower percent similarity of amino acid sequence with that of nucleotide sequence in the *Acanthaster *species and the evolution of *ATP8 *genes in asteroid species.

### Gene initiation and termination

In the case of *A. planci*, 10 of the 13 protein-coding genes use ATG as an initiation codon. Merely *ND3 *and *ND4L *genes use ATT whereas *ND1 *gene uses GTG. All initiation codons of *A. brevispinus *are the same as those of *A. planci*. The initiation codon ATG of *ND5 *gene in the *Acanthaster *species mtDNA changes to GTG in *Asterina pectinifera. *Most of the *A. planci *and *A. brevispinus *mtDNA genes terminate with a TAA codon, however *ND1*, *ND2 *and *ND6 *genes terminate with ATG. *ND4 *gene of *A. brevispinus *terminates with ATG, however, those of *A. planci *and *A. pectinifera *terminate with TAA. There is only one T residue between *CO2 *or *Cytb *genes and their downstream tRNA genes in the *Acanthaster *species and *A. pectinifera *mtDNA genomes. Furthermore, *CO1 *gene in the *Acanthaster *species terminates only with TA residue, whereas the *A. pectinifera *counterpart has a triplet codon (TAA), although *CO1 *gene is the most conserved gene among the protein-coding genes [see additional file 4]. Mitochondrial genes commonly employ several alternatives to ATG as an initiation codon and to TAA or TAG as a termination codon [13,20]. However, the mechanism by which these variations occur is not yet well understood [21]. As demonstrated in the human mtDNA genome [20], we speculate that incomplete termination codons such as T and TA are converted to canonical termination codon TAA after the cleavage of the tRNA gene by ribonuclease.

### tRNA and rRNA genes

**Figure 1:**
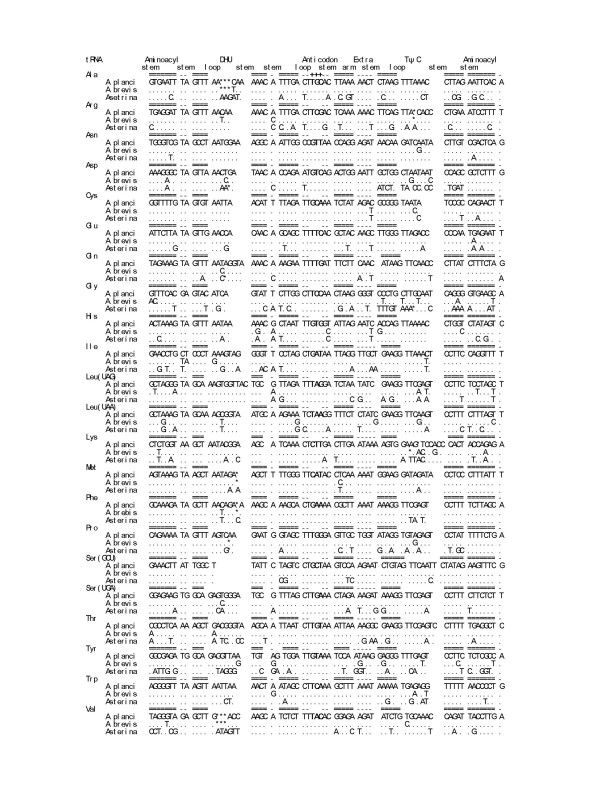
**Alignment of the twenty-two tRNA form mitochondrial genome of *A. planci*, *A. brevispinus *and *Asterina pectinifera***. * Data from Asakawa *et al*. 1995^10^. A. planci: *Acanthaster planci*; A. brevis: *Acanthaster brevispinus*; Asterina: *Asterina pectinifera*, Each anticodon is located at the middle of anticodon loop (+++). Bars above alignments indicate unambiguously aligned stem regions. Asterisks indicate deletions and dots indicate the same nucleotides as *A. planci*. The last one letter is one nucleotide protrusion of 3' amino-acid accepter stem.

Twenty-two tRNA genes are identified in *A. planci *and *A. brevispinus *mtDNA genomes by sequence comparison with known echinoderm tRNA genes and the deduced potential cloverleaf structures are shown in Figure 1.

The tRNA genes ranged in size from 67 (*tRNA*^*Cys*^) to 74 (*tRNA*^*Thr*^) and they are large enough for encoded tRNAs to fold into cloverleaf structures. As often found in other metazoan mtDNA genomes, *tRNA*^*Ser *^(GCU) lacks the DHU loop. The potential secondary structures of tRNAs are quite similar between *A. planci *and *A. brevispinus*, whereas the comparison between the *Acanthaster *species and *A. pectinifera *reveals that several size variations have occurred at the DHU and TψC loops. The comparison between the DHU and TψX loops shows that size variations are more likely to be found in the DHU loop than the TψX loop.

The echinoderm mitochondrial rRNA genes are located separately from each other [22], and this arrangement appears also in *A. planci *and *A. brevispinus *mtDNA genomes. The 5' and 3' end of *12S rRNA *genes are defined as immediately adjacent to the ends of the flanking genes *tRNA*^*Phe *^and *tRNA*^*Glu*^, in the two *Acanthaster *mtDNA genomes. However, an alignment of *A. pectinifera *does not support their 3' end boundaries; the *12S rRNA *sequences deduced from the sequence comparison with *A. pectinifera *overlapped with the beginning of *tRNA*^*Glu *^by two nucleotides.

Although the precise ends of the *16S rRNA *transcripts cannot be determined without experimentation, the inferred boundaries were determined by sequence comparison with *Pisaster ochraceus *[22] [NCBI: NC-004610]. In contrast to a rather uniform distribution of nucleotide differences over the entire protein-coding genes, although the substitution was mainly seen in the third positions, replacement differences of rRNA genes are dispersed in clusters. This variability is due to the secondary structures of rRNA genes; the stem regions are relatively conservative, whereas the loop sequences are more variable as is the case of tRNAs. Comparing with *12S rRNA *genes, *16S rRNA *genes of asteroid species seem to have more variable sites. This variability may be due to the potential loop structures that have evolved much faster than other regions in rRNAs.

### Unassigned Sequence

Intergenetic regions represent 4.6% of *A. planci *mtDNA genome and 4.7% of *A. brevispinus *mtDNA genome. The largest unassigned regions (the putative control region) are adjacent to *16S rRNA *and *tRNA*^*Thr*^. The inferred boundaries determined by sequence comparison with *P. ochraceus *suggest that this region of *A. planci *spanned 532 bp and that of *A. brevispinus *spanned 552 bp. This region contains a stem-loop structure, which is the putative origin of light-strand replication. This structure is observed close to *tRNA*^*Thr*^, and the lengths are 29 bp for *A. planci *and 34 bp for *A. brevispinus. *There is a 12 bp stem (TAACCCCCCCCC) and a 5 bp loop (CCTCC) in *A. planci*, and a 12 bp stem (ATTACCCCCCCC) and a slightly longer 10 bp loop (CCCCCCCTCC) in *A. brevispinus*. The second largest unassigned region in length is 51 bp in *A. planci *and 58 bp in *A. brevispinus *and it is located between the *ND5 *and *ND6 *genes. There are neither stem and loop structures nor promoter-like sequences in these short sequences. However, these sequences are as variable as the largest unassigned sequences. In *A. pectinifera *mtDNA genome, the second largest region is found between *ND4 *and *tRNA*^*His*^, which extends to 140 bp long, whereas in the two *Acanthaster *species it is only 8 bp long.

Aside from the large two regions, there are 4 TATATATATAA-like sequences found between the following genes in both *Acanthaster *species: *tRNA*^*Leu *^and *tRNA*^*Gly*^, *tRNA*^*Pro *^and *CO1*, *CO3 *and *tRNA*^*Pro*^, *ND6 *and *Cytb*. Except for the region between the genes for *tRNA*^*Leu *^and *tRNA*^*Gly*^, all other TATATATATAA-like sequences are located in the boundaries where the coding strand shifts to the complimentary one. As already pointed out by Asakawa *et al*. [13], these sequences are the possible bi-directional promoters, which play the role for replication and/or transcription. However, the sequence located between *16S rRNA *and *ND2 *that were reported in *A. pectinifera *and sea urchin species, *Strongylocentrotus purpuratus *[23] and *Paracentrotus lividus *[24] are not found in the two *Acanthaster *species.

### Phylogenetic relationship

**Figure 2:**
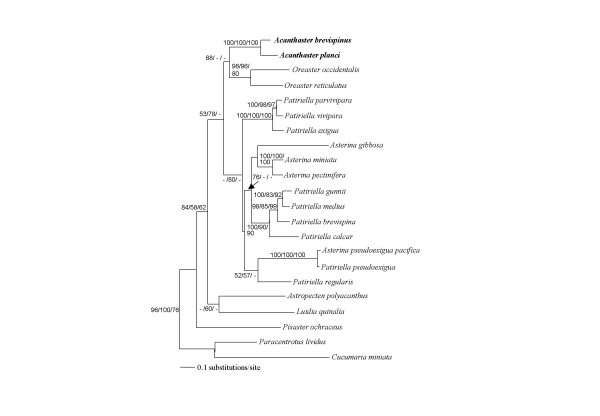
**The molecular phylogeny of asteroid species based on the *CO1 *sequence data**. *Cucumaria miniata *(Cucumariidae) and *Paracentrotus lividus *(Echinidae) were used as the out-groups. The ML tree was reconstructed with the TrN + I + G model using PAUP 4.0b10. Only bootstrap values of 50% or higher are shown to the left of internal nodes. The first numbers are from neighbor-joining analyses (1000 replications), the second numbers are from maximum likelihood analyses (100 replications) and the last numbers are from Parsimony analyses (1000 replications; characters were unweighted).

The complete *CO1 *sequences of *A. planci *and *A. brevispinus *were subjected to the ML, MP and NJ analyses together with the representative asteroid sequences [see additional file 5] available in GenBank (Figure 2). These three analyses produced almost the same tree topologies. The *CO1 *phylogeny indicated that *Pisaster ochraceus *(Forcipulatida, Asteriidae) was the most primitive taxon among asteroids. In these trees, the close affinities of species belonging to the same family (except for Asterinidae), showing monophyly, were well supported. In contrast, the confidence values for the branches linking different families were not so higher. The close affinity between *Patiriella *and *Asterina *species was found and the Asterinidae mixed together in the same group, suggesting a non-monophyletic relationship. *Acanthaster *species were positioned closer to *Oreaster *species than any other members in Valvatida, suggesting the close genetic affiliation, although the bootstrap values were not so high and these were separated from *Patiriella *and *Asterina *species by relatively high bootstrap supports in the ML method. *A. planci *and *A. brevispinus *were the closest species showing 89.2% homology between the two sequences, but these were consistently separated by 100% bootstrap supports in the tree. *Luidia quinalia *and *Astropecten polyacanthus *were grouped together and separated from *Acanthaster*, *Oreaster*, *Patiriella *and *Asterina *species. In the asteroid phylogeny using mitochondrial *12S rRNA *and *16S rRNA *also, the monophyly of each family was well supported [25,26] but the monophyly of Asterinidae was not clearly indicated from their ML and MP analyses as well as our data. In this study, *P. ochraceus *(Forcipulatida, Asteriidae) was the most primitive taxon among the asteroids. On the other hand, the mitochondrial *12S rRNA *and *16S rRNA *phylogenetic trees [26] do not support the early divergence of Asteriidae such as *Asterias amurensis *and *Coscinasterias acutispina*. Our data suggested the close affinity between *Luidia *and *Astropecten *species (both are Paxillosida), however, [26] reported a contradictory result that *Astropecten *species were not positioned close to *Luidia *species but grouped *Leiaster leachii *and *Certonardoa semiregularis *(Valvatida). The reason, why the different topology between *CO1 *and mitochondrial *12S rRNA *and *16S rRNA *sequences was obtained, is unclear. The phylogenetic relationships among asteroids remain extremely controversial in spite of many morphological and molecular studies that have been applied to this issue [26].

### Applications of mtDNA sequence of *A. planci*

*A. brevispinus *is a very rare species and was once considered as only as a short-spined individual of *A. planci *[27]. However, Birkeland & Lucas [1] and Lucas & Johns [28] reported that *A. planci *and *A. brevispinus *are genetically compatible to the extent that fertile F1 hybrids were produced. However, F2 hybrids and backcrosses were of poor viability and some of them showed morphological abnormalities, suggesting that there are barriers to introgression of genetic material between the species.

Protein and DNA technologies have provided strategies for identification when morphological features are of limited value [29,30]. A method to identify *A. planci *larvae using monoclonal antibodies provided by Hanna *et al. *(see Birkeland & Lucas 1990[1]) is not perfect because the monoclonal antibodies do not ensure discrimination among coral reef asteroids other than *Culsita novaeguineae. *As mentioned in the background of this study, a species-specific genetic marker of *A. planci *is essential to reveal the larval ecology in the field study. Recent investigations have suggested the feasibility of creating identification systems reliant on the analysis of sequence in small segments of DNA [31]. Hebert *et al*. [32] focused this discussion by proposing that a DNA barcoding system for animal life could be based upon sequence diversity in cytochrome *c *oxidase subunit 1 (*CO1*). They summarized *CO1 *sequence divergences for 13,320 congeneric pairs of animal species belonging to 11 phyla. Most pairs (79%) showed greater than 8% sequence divergence and more than 98% of species pairs showed greater than 2% sequence divergence in their study (10.9 ± 4.9% (mean ± s.d.%, n = 86) in Echinodamata). Therefore, most congeneric species pairs of animals showed enough sequence divergence to ensure their easy diagnosis, because intraspecific divergences are rarely greater than 2% and most are less than 1% [33]. In the comparison of a complete *CO1 *sequence of *A. planci *with the genetically closest species *A. brevispinus*, the sequence convergence was 10.8%. The phylogenetic tree inferred from the complete *CO1 *sequences also clearly showed the high divergence among asteroid species. In addition, no sequence variation of the partial *CO1 *gene (from 4691 bp to 5401 bp) was confirmed among the 3 *A. planci *specimens collected from different localities (data not shown). Accordingly these results indicate that the identification system for asteroid species based on the *CO1 *gene will be highly effective and it will easily provide the effective regions for species-specific markers. Moreover, universal primers for metazoan invertebrates in the *CO1 *genes were already provided [34], therefore, PCR-RFLP (Restriction Fragment Length Polymorphism) assay can also be applied as another tool for species identification of *A. planci.*

For polymorphic marker using mtDNA variation, the desirable characteristics are as follows: evolutional rate is rapid enough to distinguish among local populations and substitutions are neutral. In *A. planci *mtDNA sequence, the largest unassigned region between *tRNA*^*The *^and *16S rRNA *is most variable. In this region, size variations ranging from 538 to 546 bp are observed in the 23 individuals. The variation includes 62 base substitutions and 5 deletions/insertions ranging from 1 to 5 bp (data not shown). These sequences can be useful in the analysis of intraspecific variation of *A. planci *collected from several localities.

## Conclusion

The complete mitochondrial genome sequences of *Acanthaster planci *and its sibling, rare species, *A. brevispinus *were determined and compared with another known asteroid species, *Asterina pectinifera*. The genome contains 37 genes most commonly found in metazoan mtDNA and they are in the same order as in *A. pectinifera*. Characteristics of the two *Acanthaster *species are quite similar when compared with *A. pectinifera*, though there exist some significant differences between them especially in the unassigned regions. The sequence information will be useful for molecular ecological studies of *A. planci *in future.

## Methods

### Animal samples and DNA Extraction

A specimen of *Acanthaster planci *for complete mtDNA sequencing was collected at Akajima Island, Okinawa Prefecture, Japan in August 2003 by snorkeling. A specimen of *A. brevispinus *was collected at an offshore site at Kushimoto, Wakayama Prefecture, Japan in October 2003. Twenty-three specimens of *A. planci *to observe the variability of the largest unassigned sequence were collected from Ishigaki Island (n = 4), Tokashiki Island (n = 10) in Okinawa Prefecture and Kinko Bay (n = 9) in Kagoshima Prefecture, Japan. The total genomic DNA of *Acanthaster *species was extracted from the tube feet using a DNeasy kit (Qiagen) following the manufacturer's procedure.

### Long PCR and DNA sequencing

Oligonucleotide primers were designed in accordance with sequences reported in *Asterina pectinifera *[12] and other species in order to amplify the intervening regions. Sequences were completely determined from the ends of these PCR fragments, then internally by primer walking.

Three partial sequences of *A. planci *and *A. brevispinus *mtDNA were obtained. They were amplified to a total volume of 100 μl containing 5 U of LaTaq (TaKaRa), 50 μl of 2 × GC buffer I, 2.5 mM of MgCl_2_, 16 μl of dNTPs mixture (2.5 mM each), 0.5 μM of each oligonucleotide primers, and 10 to 50 ng of the genomic DNA. The mixture was subjected to 40 cycles of amplification in a thermal cycler (PC-800, ASTEC). The oligonucleotide primers of *A. planci *and *A. brevispinus *are summarized in Table 6 [see additional file 6]. The first cycle was preceded by initial denaturation for 1 min at 94°C. Each cycle consisted of denaturation for 30 sec at 94°C, annealing for 30 sec at 50–55°C, and extension for 7 min. The last cycle was followed by a final extension step for 10 min at 72°C.

All PCR products were electrophoresed in 1% agarose gels and stained with ethidium bromide. The agarose gel slices containing the PCR-product band were excised under UV, and DNA was purified from the agarose plug by using GenEluteTM Agarose Spin Columns (SIGMA), followed by an ethanol precipitation. Amplified products were either sequenced directly or cloned into pGEM-T^® ^easy vector in the pGEM-T^® ^easy system (Promega) and TopoXL^® ^vector using the TopoXL (Invitrogen). Recombinant plasmids were extracted by the QIAprep Spin Miniprep kit (Qiagen).

All DNA sequence data were determined using ABI PRISM 3100 Genetic Analyzer (Applied Biosystems) DYEnamic ET Terminator Cycle Sequencing Kit and DYEnamic Terminator Dilution Buffer (Amersham Bioscience).

### Phylogeny

Complete *CO1 *sequences, excluding primer regions, of *A. planci *and *A. brevispinus *were aligned with 18 other asteroid sequences obtained from GenBank using the ClustalX package [35] and edited manually. *Cucumaria miniata *(Cucumariidae) and *Paracentrotus lividus *(Echinidae) were used as the out-groups [see additional file 5]. A total of 1551 bp sequence were analyzed using maximum likelihood (ML), maximum parsimony (MP) and neighbour-joining (NJ) methods in PAUP* 4.0b10 [36]. In order to find the best model for the *CO1 *sequences we applied Modeltest (version 3.07) [37]. The best-fit model was TrN + I + G [38] with assumed proportion of invariable site = 0.5180, gamma distribution shape parameter = 0.6099 and base substitution categories (AC = 1.0000, AG = 10.3590, AT = 1.0000, CG = 1.0000, CT = 8.7274, GT = 1.0000). Base frequencies were set at A = 0.3725, C = 0.2639, G = 0.0884 and T = 0.2753. ML analyses were performed with the heuristic search option starting with NJ and TBR branch swapping. One hundred replicates were performed in ML bootstrap analysis. In MP 100 random additions were done using the heuristic search option and TBR branch swapping. Characters were unordered and weighted equally. Bootstrap analyses with 1000 replicates were conducted. The best-fit model suggested by Modeltest (see above) was also used to compute dissimilarity values. The distance matrix obtained was used to build a tree with NJ. One thousand replicates were performed in NJ bootstrap analysis.

## Abbreviations

*CO1, CO2, CO3*, cytochrome oxidase subunit I, II and III protein genes; *Cytb*, cytochrome b gene; *ATP6*, *ATP8*, ATP synthase subunit 6 and 8 genes; *ND1, ND2*, *ND3, ND4, ND4L, ND5 *and *ND6*, NDAH dehydrogenase subunit 1–6, 4L genes; *12S rRNA *and *16S rRNA*, small and large ribosomal RNA genes; tRNA, transfer RNA genes with standard 3-letter 22 amino acid abbreviations are used. PCR-RFLP, Polymerase Chain Reaction based – Restriction Fragment Length Polymorphism.

## Authors' contributions

KN and MH were primarily responsible for the design, coordination and conducting the study. NY and MS were responsible for determining and assembling the mtDNA sequences. NY drafted the manuscript and figures, and MH contributed suggested some valuable comments. KN extensively revised the manuscript. SN conducted phylogenetic analysis. This research has been financially supported by JSPS (The Japan Society for the Promotion of Science), Grand-in-Aid for Scientific Research (A) (No. 14205071 and No. 15254002), and by The Cabinet Office of Japan through Research Institute of Subtropics. KN is the project leader for these grants. All authors have read and approved the final manuscript.

## Supplementary Material

Additional File 1 **Table 1: Localization of genes and features in the mitochondrial genome of *Acanthaster planci *and *A. brevispinus***. Genes with asterisks are coded on the complimentary H strand. Click here for file

Additional File 2 **Table 2: Comparison of base composition of the mitochondrial genomes of *Acanthaster planci***, ***A. brevispinus *and *Asterina pectinifera***. * Data from Asakawa *et al. *[10]. Click here for file

Additional File 3 Table 3: Total codon usage in the 13 protein-coding genes of *Acanthaster planci*, *A. brevispinus *and Asterina pectinifera. *Data from Asakawa *et al. *[10]. Click here for file

Additional File 4 **Table 4: Comparison of nucleotide and amino acid sequences in 13 protein-coding genes of *Acanthaster planci*, *A. brevispinus *and *Asterina pectinifera.***Genes that showed size variations are indicated as bold. Click here for file

Additional File 5 **Table 5: Primer sequences for long PCR to amplify the mitochondrial DNA in *A. planci *and *A. brevispinus.*** Click here for file

Additional File 6 **Table 6: List of specimens used for phylogenetic analysis.** Click here for file
